# Examining the Efficacy of Drain Tip Cultures in Predicting Postoperative Surgical Site Infections in Hip Arthroplasty: A 15-Year Retrospective Study

**DOI:** 10.7759/cureus.46395

**Published:** 2023-10-03

**Authors:** Akira Toga, Ayush Balaji, Osamu Hemmi, Ken Ishii, Shigeyuki Tokunaga, Shojiro Katoh, Ryoichi Izumida

**Affiliations:** 1 Department of Orthopaedic Surgery, Edogawa Hospital, Tokyo, JPN; 2 Department of Orthopaedic Surgery, Keio University School of Medicine, Tokyo, JPN; 3 Medicine, Hull York Medical School, York, GBR; 4 Keiyu Artificial Joint Center, Edogawa Hospital, Tokyo, JPN; 5 Spine Surgery, Society for Minimally Invasive Spinal Treatment (MIST), Tokyo, JPN; 6 Department of Orthopedic Surgery, Edogawa Hospital, Tokyo, JPN

**Keywords:** prognostic value, surgical drain, revision hip arthroplasty, primary hip replacement, postsurgical infection, total hip arthroplasty (tha), surgical site infection, drain tip culture, bipolar hip arthroplasty (bha)

## Abstract

Background

Postoperative surgical site infections (SSIs) are a significant complication of surgical procedures, leading to increased morbidity, prolonged hospital stays, and substantial healthcare costs; however, the use of drain tip cultures to diagnose SSIs in patients is controversial. The objective of this study was to evaluate the efficacy of drain tip cultures for the prediction of postoperative SSIs in patients recovering from hip arthroplasty.

Methodology

The data were collected from 1204 patients who underwent hip arthroplasty procedures over 15 years, and statistical analysis was performed to evaluate the diagnostic value of drain tip culture in determining surgical site infection. We also used these data to evaluate whether preexisting conditions such as hypertension or diabetes affected the probability of a patient getting an SSI.

Results

Drain tip cultures were positive in 12 of 1,112 cases of primary hip arthroplasty, but only one of these 12 patients was ultimately diagnosed with an SSI (sensitivity, 12.5%; specificity, 99.0%; p = 0.0834). Results from postoperative drain tip cultures performed in patients undergoing revision arthroplasty included two false positives and three false negatives; interestingly, no true positives were detected in any of the revision arthroplasty cases we evaluated (sensitivity, 0％; specificity, 97.8％; p = 0.9355).

Conclusion

Our results indicate that drain tip cultures have no statistically significant predictive value for the diagnosis of postoperative SSIs and thus should not be used as a primary diagnostic or predictive tool for SSIs. We recommend exploring other diagnostic tools for the postoperative diagnosis of SSIs. Standardized guidelines should therefore be established to improve the predictive value of the different methods.

## Introduction

Surgical drains are traditionally inserted after total hip arthroplasty (THA) and bipolar hip arthroplasty (BHA) procedures to prevent the formation of a hematoma at the surgical site. Bacterial cultures from the tips of the drainage tubes are routinely performed after drain removal, but the efficacy of such drain tip cultures for detecting and diagnosing postoperative surgical site infection (SSI) remains unclear. SSIs are perioperatively diagnosed using samples taken from the synovial capsule. Although the incidence of SSI after hip arthroplasty is between 1% and 3%, the complications of SSIs associated with this procedure are often severe and require immediate intervention [1-3]. It is therefore important to evaluate the effectiveness of the diagnostic tools and protocols used to diagnose SSI after hip arthroplasty.

Several previous studies have documented specific correlations between the results of drain tip cultures and the incidence of SSIs after total knee arthroplasty and spinal surgery [4]. However, these studies included a small number of patients and drain tip samples, and thus the likelihood of statistical errors was high. Petsatodis et al. [5] determined that a minimum of 1,000 cultures would be required for an accurate conclusion regarding the efficacy of drain tip cultures for diagnosing SSIs after hip arthroplasty. Because only one study to date has achieved such a large sample size, the efficacy of drain tip cultures remains controversial. Achieving a clear conclusion therefore requires increasing data availability while maintaining standard surgical procedures.

This study aimed to evaluate the effectiveness of drain tip cultures for predicting and diagnosing SSIs in patients who underwent hip arthroplasty, intending to obtain a large enough sample size for a robust and accurate assessment. To that end, we performed a retrospective analysis of drain tip cultures from 1,087 primary THA patients, 25 primary BHA patients, and 92 patients who underwent revision arthroplasties between 2005 and 2019 at Edogawa Hospital in Japan. From a literature search, we found multiple studies that discussed the correlation between drain tip culture results and the occurrence of surgical site infection [5-7]. Results from previous studies have varied, and controversies persist in the published literature, indicating a gap in our understanding of the topic. Given this backdrop, the primary objective of our retrospective investigation was to assess the predictive potential of drain tip culture in determining SSI following THA with an adequate sample size.

This article was previously posted to the Research Square preprint server on August 24, 2022.

## Materials and methods

We performed a retrospective evaluation of findings from 1,112 of the 1,159 patients who underwent primary hip arthroplasty at our hospital over the past 15 years (2005-2019) and 92 patients who underwent revision hip arthroplasty. The data were collected from electronic medical records. Forty-seven cases that did not include records of drain tip cultures were eliminated from further consideration. Patients were between 25 and 94 years of age (average: 65.9 years) and included 232 males (20.8%) and 880 females (79.1%). The primary indications for hip arthroplasty included osteoarthritis (895 cases, 80.5%), idiopathic necrosis of the femoral head (102 cases, 9.2%), and other conditions including rheumatoid arthritis and osteonecrosis (115 cases, 10.3%). 105 patients with diabetes, 462 patients with hypertension, and 654 patients with an abnormal body mass index (BMI) above 25 or below 18.5.

This study (approval number: 200406011) was approved by the institutional review board at Edogawa Hospital and was performed in accordance with the Helsinki Declaration. All study participants provided informed consent prior to being enrolled in the study.

All surgical procedures were performed using either a posterior approach or an anterolateral supine approach by one of two surgeons using the same aseptic techniques. Specifically, we used the same asepsis protocol and antibiotic regimen in all cases, the same range of implant types, and a standardized drain removal protocol to control for as many variables as possible. The asepsis protocol for these procedures included preoperative scrubbing with iodopovidone (Betadine) and draping. Cefazolin (1.0 g) and Amikacin (200 mg) were administered intravenously 30 minutes before surgery and then every 12 hours for three days after surgery. Cephalexin (250 mg) was taken orally four times a day for the subsequent seven days. Due to an abundance of high-risk patients, such as an increased smoking population, diabetics, and geriatric patients, the standardized antibiotic protocol included an extended prophylaxis period [8].

Surgical sites were cleaned with pulsed lavage both before and after implantation of the prosthesis. The fascia was closed using a standard closure technique. Before closure, the operative site was copiously irrigated with iodopovidone, which was also reapplied to the incision site using a sterile sponge. A 3-mm-diameter silicone catheter was inserted under the fascia lata at the time of closure to promote drainage and infused with a solution of 10 mL 0.75% anapeine, 10 mL 10% tranexamic acid, and 40 mg gentamicin. The sterile technique was maintained through the insertion and removal of the catheter. The wound was covered using a water- and film-proof dressing with a transparent hydrogel pad. The closed suction drainage system clamped shut during surgery, was opened two hours after surgery to allow for adequate suction, and was eventually removed 48 hours after surgery.

After removal, the catheter was cut approximately 2 cm from the tip and submitted to the laboratory for culturing. Catheter samples were submitted in a sterile spitz with saline and then centrifuged for 10 minutes at 3500 rpm. The supernatant was decanted after centrifugation, and the sample was smeared onto chocolate agar (carbon dioxide culture), liquid agar (aerobic culture), blood agar (anaerobic culture), bromothymol blue agar (aerobic culture), and Gifu anaerobic semi-fluid medium. All cultures were incubated at 37 °C for 24 hours. Samples with visible bacterial growth were then Gram-stained, and samples without visible growth were cultured for one extra day. Additional biochemical tests to identify specific bacterial species were performed on any culture that produced visible bacterial growth. Samples in which no bacterial growth was observed even after an additional day of culturing were considered negative for infection. All patients were followed up at 3 months, 6 months, and 12 months for any signs of infection, as well as annually following this time period.

To validate the results from the tip cultures, surgical sites were inspected post-operatively for signs of SSI by a healthcare staff member according to guidelines from the Centers for Disease Control (CDC) [9].

We improved upon the CDC guidelines by diagnosing an SSI based on (1) the isolation of a bacterial pathogen in conjunction with (2) local pain or tenderness, swelling, redness, heat, and (3) elevated serum levels of C-reactive protein (CRP). In cases where the diagnosis remained unclear, we consulted multiple physicians and reached a collective decision, ensuring the diagnosis's reliability and decreasing the chance of a misdiagnosis.

Results from all patients in this 15-year study were compiled in Microsoft Excel® spreadsheets and analyzed for evidence that was part of our criteria. The language used in the spreadsheet was consistent across all patients and agreed upon by the research team and the inputting physician. We calculated the correlation between positive drain tip cultures and SSI diagnoses using a one-tailed Fisher's exact test with a confidence interval of 95% (i.e., the null hypothesis was rejected if p < 0.05). We also calculated the positive predictive value, which measures the effectiveness of a test at predicting a particular condition. In our case, we measured whether drain tip culture can accurately predict an SSI when it occurs. We therefore used the positive predictive value as an indicator for effectiveness, as our goal was to determine the prognostic value of positive drain tip cultures for the diagnosis of SSI.

## Results

Of the 1,112 patients who underwent primary hip arthroplasty at Edogawa Hospital from 2005-2019, 12 patients had positive drain tip cultures (Table 1), and 8 patients (0.72%) were suspected of having a postoperative SSI. However, only one SSI case was associated with a positive drain tip culture. Of the seven SSI cases with negative tip culture results, four (0.36%) required washing and surgical debridement. All eight SSI cases were diagnosed as superficial. Based on these results, the sensitivity of drain tip culture for postoperative SSI was 12.5% and the specificity was 99.0%, with a positive predictive value of 8.3% and a negative predictive value of 99.4%. A Fisher exact test performed on these data showed no statistically significant correlation between drain tip culture results and the onset of an SSI (p = 0.0834).

**Table 1:** Correlation between postoperative surgical site infection and the incidence of positive drain tip cultures from patients who underwent primary hip arthroplasty from 2005 to 2019. The p-value was 0.0834 (p-value considered significant if p < 0.05), indicating that there was no statistically significant correlation. *All SSIs diagnosed in this patient cohort were superficial in nature.

	Number of patients with SSIs*	Number of patients without SSIs	p-value
Positive cultures	1	11	
Negative cultures	7	1093	
Total	8	1104	0.0834

In the single case of suspected SSI with a positive drain tip culture, the drain tip culture was positive for methicillin-resistant Staphylococcus epidermidis. The other 11 patients with false-positive drain tip cultures had no symptoms or indications of SSI, and their drain tip cultures were positive for bacteria that are part of the native skin flora, which may not result in infection. The bacterial strains detected in these false-positive drain tip cultures included S. epidermidis (n = 3), S. cohnii (n = 2), and Escherichia coli (n = 2; Table 2).

**Table 2:** Species identified in drain tip cultures from patients who underwent primary hip arthroplasty at Edogawa Hospital from 2005 to 2019. *One patient returned two species of bacteria in their culture result.

Bacteria	Number of culture-positive drain tips (total n = 12)*
S. epidermidis	3
S. cohnii	2
E. coli	2
S. haemolyticus	1
Acinetobacter iwoffi	1
Methicillin-susceptible *S. aureus*	1
Enterobacter cloacae	1
Methicillin-resistant *S. epidermidis*	1
Fungi	1

In addition to obtaining samples from patients undergoing primary hip arthroplasties, we also performed drain tip cultures on 92 patients who underwent revision hip arthroplasties. Neither of the two patients with positive drain tip cultures was diagnosed with an SSI (Table 3), resulting in a drain tip culture sensitivity of 0.0%, a specificity of 97.8%, a positive predictive value of 0.0%, and a negative predictive value of 96.7% (Table 4).

**Table 3:** Correlation between postoperative surgical site infection and the incidence of positive drain tip cultures who underwent revision hip arthroplasty from 2005 to 2019. The p-value was 0.936 (p-value considered significant if p < 0.05), indicating that there was no statistically significant correlation. *One case of SSI after revision arthroplasty was deep in nature.

	Number of patients with SSIs*	Number of patients without SSIs	p-value
Positive cultures	0	2	
Negative cultures	3	87	
Total	3	89	0.936

**Table 4:** Statistical evaluation of the use of drain tip cultures to predict surgical site infections after primary or revision hip arthroplasty.

Outcomes	Primary arthroplasty (N = 1112 patients)	Revision arthroplasty (N = 92 patients)
Sensitivity	12.5%	0.0%
Specificity	99.0%	97.8%
Positive predictive value	8.3%	0.0%
Negative predictive value	99.4%	96.7%
p-value	0.0834	0.936

Overall, there was no statistically significant correlation between the results of drain tip culture and the incidence of SSI in patients who underwent revision hip arthroplasties (one-tailed Fisher's exact test, p = 0.936). However, due to the modest sample size, Fisher's exact test may not provide a decisive conclusion regarding the effectiveness of tip culture for predicting SSI in these patients. The two positive drain tip cultures were positive for *E. coli* and *Burkholderia cepacia*, respectively (Table 5). These false-positive results suggest that detecting these bacteria, which are components of the normal skin flora, may not indicate infection.

**Table 5:** Bacteria identified in drain tip cultures from patients who underwent revision hip arthroplasty at Edogawa Hospital from 2005 to 2019.

Isolated bacteria	Number of culture-positive drain tips (total n = 2)
E. coli	1
B. cepacia	1

In order to reduce confounding bias, we observed the distribution of positive cultures throughout the data collection period. Notably, these instances were not concentrated or isolated at any specific interval. Instead, they were dispersed evenly across the entire duration of our study. This dispersion suggests that the occurrence of positive cultures was not influenced by temporal factors or specific events during the data collection phase. Alongside this, to determine whether preexisting patient factors affected the chance of developing SSI, we evaluated the incidence of SSI with various patient characteristics, including age, gender, hypertension, diabetes, steroid use, and BMI (Table 6). However, none of these factors significantly correlated with developing an SSI.

**Table 6:** Incidence of positive drain tip cultures in relation to patient characteristics and preexisting conditions. No statistically significant correlations were observed. All p-values are considered significant if p < 0.05.

		SSI	No-SSI	p-value
Gender	Male	3	229	0.2223
	Female	5	875	
Age	Above 65	4	588	0.5143
	Under 65	4	473	
Hypertension	Hypertensive	4	458	0.4188
	Non-hypertensive	4	673	
Diabetes	Diabetic	1	104	0.5583
	Non-diabetic	7	973	
BMI	Normal BMI	4	476	0.4603
	Abnormal BMI	4	650	
Steroid use	Steroid user	0	21	0.8558
	Non-steroid user	8	1064	

## Discussion

There has been much recent debate on the utility of drain tip cultures for predicting and diagnosing postoperative SSIs. Studies published before 2004 concluded that drain tip culture results were positively correlated with SSI diagnoses in cases of total hip arthroplasty. However, these studies all had small sample sizes (approximately 100 patients), making the conclusions of these retrospective analyses controversial, as small sample sizes can sometimes lead to fallacious results.

Our study, which included 1,112 patients who underwent primary hip arthroplasty, agrees with previous studies that do not support using routine drain tip culture after hip replacement to diagnose postoperative infection [5,6,10]. The incidence of SSI in our study aligns with the internationally reported literature. The low positive predictive value (3.3%) suggests that the prognostic value of drain tip cultures is minimal, the likelihood of false-negative results is high, and, overall, that it is ineffective for predicting or diagnosing an SSI in postoperative patients.

The false-positive results in the data could result from contamination during drain tip removal, transport, or culturing. Drain tip contamination has not been emphasized in other studies; however, based on the type of bacteria we found and the lack of clinical findings indicative of an infection, we suggest that drain tip contamination is a possible cause of false-positive culture results. Relying on the results of drain tip cultures could lead to the unnecessary use of antibiotics, thus increasing the risk of side effects and healthcare costs. Skin flora contamination may have contributed to the findings reported in all similar studies published to date [11,12]. The length of time that the drain remains at the operative site could also affect the likelihood of developing an SSI and the risk of skin flora contamination of the drain tip culture [13]. It is therefore essential to standardize the time the drain is removed.

Based on the low prognostic and diagnostic value of drain tip cultures, we recommend that alternate techniques be evaluated for the prediction and diagnosis of SSIs. It is critical to recognize that there was a high monetary cost as well as time loss in performing the 1,204 drain tip cultures, with positive results incurring additional costs. The costs associated with drain tip cultures will increase as the number of people undergoing hip arthroplasty increases, raising additional questions about the feasibility of drain tip cultures.

We also evaluated the incidence of SSI with patient age, gender, BMI, and history of hypertension, diabetes, and steroid use. Age, gender, and the tested comorbidities were not statistically significantly correlated with the development of SSI. Understanding the relationships between patient comorbidities and the incidence of SSI may allow for patient-specific SSI screening procedures to be introduced, which may prove better than drain tip cultures. There could also be several other potential risk factors for SSIs after hip arthroplasty, including patient-related factors such as rheumatoid arthritis, malnutrition, smoking, and alcohol consumption. Surgical factors such as the duration of the surgery, blood transfusions, and intraoperative complications can also influence the risk of SSIs. Additionally, postoperative factors like prolonged wound drainage, hematoma formation, and early mobilization may also play a role. Future studies could aim for a more comprehensive evaluation of all potential risk factors to provide a clearer understanding of the predictors of SSIs after hip arthroplasty.

There are limitations associated with all the studies considered here, including the present study. The first is that all studies were retrospective, although in our case, using a standard and uniform clinical protocol throughout the study period mitigated potential confounding factors associated with variation among surgeons or procedures. All SSIs diagnosed in patients undergoing primary hip arthroplasty were superficial, meaning that the infection only involved the skin or subcutaneous tissue and did not extend down to the muscle, fascia, or organ space. We identified only one case of deep tissue infection in a patient who underwent revision hip arthroplasty. Therefore, our results are unclear on whether drain tip cultures effectively predict deep tissue or organ/space SSIs. We recognize that it is difficult to compare across studies because of variations in postoperative antibiotic protocols, asepsis techniques, implant types, and surgical approaches. After collecting samples over a 15-year period, we tried to ensure a diverse representation of patient demographics. As with all studies, there are potential limitations in generalizing our findings, as mentioned above, such as variations in different centers' approaches to the method of drain removal and the time of culture result.

Other problems include the lack of universal guidelines for diagnosing and preventing SSIs and the additional expense of drain tip cultures, which may act as a barrier to their clinical use [14]. Although the Enhanced Recovery After Surgery (ERAS) protocol is increasingly being adopted, many hospitals still use closed suction drainage catheters following hip arthroplasty [15]. In addition, given the low incidence of SSI associated with hip arthroplasty procedures, an ideal study would include a substantially larger patient cohort in which many patients tested positive for SSI. Given the present studies and the general lack of statistical support for the routine use of drain tip cultures, other diagnostic tools should be explored as potential alternatives. Such tools may include screening for hematoma, as previous studies have observed a correlation between the incidence of hematoma and SSIs, or diagnostic tools such as physical examination [16].

In the context of drain tip cultures and their reliability, it is imperative to prioritize the prompt treatment of infections in patients exhibiting clinical signs of infection, irrespective of the results obtained from drain tip cultures. This should be guided by clinical examination and guidelines regarding diagnosis for surgical site infection, such as those mentioned by the CDC listed above, as well as the clinical judgement of whether there is a high probability of active infection. Further action may include, but not be limited to, antibiotic use, washout of the site, debridement, and even revision of the arthroplasty based on the extent of the infection.

## Conclusions

Drain tip cultures are routinely used in hip arthroplasty but also in spinal procedures and knee arthroplasty. Continuing to evaluate the effectiveness of drain tip cultures is vital for the broader field of orthopedic surgery. Some hospitals within Japan and other countries continue to utilize drain tip cultures as a diagnostic tool to detect SSI; however, based on the results of our study as well as a review of the existing literature, drain tip cultures do not have a high enough positive predictive value to be used as an effective diagnostic tool, and clinical guidance should be built off these conclusions.

Furthermore, relying on drain tip cultures for diagnosing SSIs can lead to the unnecessary use of antibiotics, which not only increases the risk of side effects but also escalates healthcare costs. Considering the high monetary cost and time investment associated with performing a large number of drain tip cultures, it raises questions about the feasibility and cost-effectiveness of using this diagnostic method, especially as the number of hip arthroplasty procedures continues to rise. Given the current evidence and the lack of robust statistical support for drain tip cultures, orthopedic surgeons and healthcare institutions should reconsider their clinical practices and explore other diagnostic tools as potential alternatives. Future investigations may focus on developing more accurate diagnostic tools.
